# Glucose metabolism outcomes in acromegaly patients on treatment with pasireotide-LAR or pasireotide-LAR plus Pegvisomant

**DOI:** 10.1007/s12020-021-02711-3

**Published:** 2021-04-27

**Authors:** Sabrina Chiloiro, Antonella Giampietro, Felicia Visconti, Laura Rossi, Federico Donfrancesco, Cara M. Fleseriu, Federica Mirra, Alfredo Pontecorvi, Andrea Giustina, Maria Fleseriu, Laura De Marinis, Antonio Bianchi

**Affiliations:** 1 Pituitary Unit, Department of Endocrinology and Diabetes, Fondazione Policlinico Universitario A. Gemelli, IRCCS, Rome, Italy; 2 Dipartimento di Medicina e Chirurgia Traslazionale, Università Cattolica del Sacro Cuore, Rome, Italy; 3 Pituitary Center, Oregon Health and Science University, Portland, OR USA; 4 University of Pittsburgh, Pittsburgh, PA USA; 5 Institute of Endocrie and Metabolic Sciences, Vita-Salute San Raffaele University and IRCCS San Raffaele Hospital, Milano, Italy

**Keywords:** Diabetes, Glucose intolerance, Impaired fasting glucose, Impaired glucose tolerance, Somatostatin analogues, Growth hormone receptor antagonist

## Abstract

**Introduction:**

Disorders of glucose metabolism are a serious acromegaly comorbidity and may be differently impacted by medical treatments of acromegaly. In this retrospective longitudinal multicenter study, we investigated the outcome of glucose metabolism and its predictors in patients treated with Pasireotide LAR (PAS-LAR) alone or in combination with Pegvisomant (PAS-LAR + Peg-V).

**Subjects and methods:**

Acromegaly patients treated continously with PAS-LAR or PAS-LAR + Peg-V for at least 6 months.

**Results:**

Forty patients (25 females, 15 males) were enrolled. At last visit, 27/40 patients (67.5%) reached biochemical control of acromegaly. Overall, glucose metabolism improved in 3 (all in PAS-LAR + Peg-V; 7.5%), worsened in 26 (65%) and remained unchanged in 11 patients (27.5%). Glucose metabolism worsened in 25 patients (73.5%) treated with PAS-LAR and in a single patient (16.7%) treated with PAS-LAR + Peg-V (*p* < 0.001). Among patients treated with Pas-LAR alone, GH at baseline was higher in those with worsening of glucose metabolism (*p* = 0.04) as compared to those with stable glucose status. A significantly higher reduction of HbA1c was observed in patients treated with PAS-LAR + Peg-V, as compared with those treated with PAS-LAR alone (*p* = 0.005).

**Conclusions:**

Our data confirmed that glucose metabolism in patients treated with PAS-LAR is often worsened, and may be predicted by entity of baseline GH hypersecretion and by the dose of PAS-LAR. Moreover, our data, although limited by small numbers, may suggest that the combination treatment PAS-LAR + Peg-V can improve glucose homeostasis in selected patients.

## Introduction

Acromegaly is a systemic disease, caused by the hypersecretion of growth hormone (GH) and type I insulin-like growth factor (IGF-I), that occur in almost all cases due to a pituitary tumor secreting GH [1]. The GH and IGF-I excess are responsible of several clinical disorders affecting multiple organ systems [2]. Diabetes mellitus and prediabetes are among the most frequent and clinically relevant comorbidities of acromegaly [3, 4].

Previous studies on glucose metabolism in acromegaly investigated the prevalence of diabetes mellitus, reported between 13 and 31.9% of cases [5–10]. Additional studies proved a prevalence of impaired fasting glucose (IFG) of 8.9% [10] and of impaired glucose tolerance (IGT) from 18.4% to 31.6% of cases [5, 7, 10]. Numerous studies have investigated the impact of different treatment regimens of acromegaly on glucose homeostasis [11–15]. However, no comparative studies have been performed and limited information on the predictors of glucose metabolism outcome with different acromegaly treatment are available. The aim of this study was to investigate the prevalence and predictors of glucose metabolism abnormalities after treatment with Pasireotide LAR (PAS- LAR) alone or in combination with Pegvisomant (Peg-V), in acromegaly patients, resistant or intolerant to first generation somatostatin receptor ligand (first gen-SRLs).

## Subjects and methods

A longitudinal, observational, retrospective multicenter study was performed on consecutive acromegaly patient with acromegaly before (baseline) and after (end of study) treatment with PAS- LAR and PAS- LAR plus Peg-V.

All patients were enrolled according to the following inclusion and exclusion criteria.

### Inclusion criteria

1. Acromegaly patients treated with first gen-SRLs for at least 6 consecutive months and considered partial/complete resistant or intolerant to SRLs [16, 17] at the study entry;
2. patients on treatment with PAS- LAR and PAS- LAR plus Peg-V, for at least 6 months;
3. patients older than 18 years;

### Exclusion criteria

1. Concomitant treatment with drugs known to influence glucose metabolism (as anti-psychotics, beta-blockers or thiazide diuretics), with the exception of glucocorticoid replacement therapy for central hypoadrenalism and anti-diabetic therapies for patients with glucose abnormalities at baseline;

### Study protocol

Demografics and patients’ clinical history were collected from records. Biochemical markers of acromegaly and glucose metabolism were collected both at baseline and at last evaluation visit (end of the study).

### Biochemical evaluation of acromegaly

Resistance to conventional SRLs was defined [16, 17]:

- partial: in case of a reduction of random GH and/or IGF-I > 50% with respect of pre-therapy values in absence of biochemical control of acromegaly disease and/or a reduction of tumor volume > 20%;
- complete: in case of a not-significant reduction of IGF-I and random GH value and/or of tumor volume.

At baseline, active acromegaly was diagnosed in patients with random GH higher than 1.0 ng/mL and IGF-I concentrations above the normal ranges for age [18]. During the study period, acromegaly patients were biochemically evaluated at least every 6 months, but variable depending on clinical status and Center’s protocol. Acromegaly was defined controlled if normal age and gender IGF-I values and random GH was below 1.0 ng/mL [19]. Patients on treatment with Peg-V were evaluated only by serum IGF-I. A mean of all serum IGF-I values was calculated for each patient in order to have an integrated measure of evaluations of during the study period. IGF-I was expressed as IGF-I for upper limit of normal (ULN), according to normative data for each center laboratory. GH and IGF-I were measured in all centers using chemiluminescent immunometric assays (Immulite 2000, Siemens Healthcare, Erlangen, Germany). The standard for GH was IS 80/505 until 2010, IS 98/574 afterwards. The standard for IGF-I was IS 02/254. Coefficients of variation were below 5% for both assays.

### Glucose metabolism

Data of glucose metabolism status were collected at baseline and at end of the study (last visit). According to medical history, fasting or after oral glucose tolerance test (OGTT) blood glucose and glycated hemoglobin (HbA1c) patients were classified as euglycemic, with IGT (impaired glucose tolerance)/IFG (impaired fasting glucose) or diabetes mellitus (DM) [20]. During follow-up, which was performed according to clinical practice, glucose evaluation was conducted every 3 or 6 months. At the end of the study (last visit), the glycometabolic status was defined:

- improved, in case of reduction/withdrawal of anti-diabetic treatments or improvement of serum glucose paramentes;
- worsened, in case of increased dosage or new prescription of anti-diabetic treatments or worsening of serum glucose paramentes;
- unchanged, in case of manteinance of the same anti-diabetic treatments or in case of not clinically significant variations of glucose parameters.

### Ethical approval

Registry databases were approved by institutional review boards of each institution and informed consent was waived for individual patients. All procedures performed in studies involving human participants were in accordance with the ethical standards and with the 1964 Helsinki declaration and its later amendments or comparable ethical standards.

### Statistical analysis

All data were expressed as mean and standard deviation. Fisher exact and Student *t* tests were used to compare categorical and quantitative un-paired data. A *P* value < 0.05 was considered statistically significant.

## Results

A total of 40 patients, 25 females (62.5%) were analyzed. Mean age at study entry was 35.4 years (SD: 11.7). The first line treatment was pituitary surgery for 36 patients (90%) and medical therapy with first generation SRLs for the remaining 4 patients (10%). All 40 patients who entered the study had undergone previous therapy with first generation SRLs. At baseline, all patients had active acromegaly with partial resistence to first generation SRL in 11 cases (27.5%) and complete resistant in the remaning 29 cases (72.5%). Thirty-four patients were treated with Pasi- LAR alone (85%) and 6 patients with Pasi-LAR plus Peg-V (15%).

At baseline, the glucose metabolism was considered normal in 18 patients (45%), 13 (32.5%) were affected by IGT/IFG and 9 patients (22.5%) by DM. Eleven patients (27.5%) were diet-controlled, 3 patients (7.5%) treated with oral hypoglycemic drugs and 6 patients (15%) with insulin. The remaining 20 patients were not treated for glucose abnormalities (50%).

The baseline clinical features of the two treatment groups are summarized in Table 1.

**Table 1:** Study population features stratified for acromegaly treatments

	Pasireotide LAR	Pasireotide LAR plus Peg-V	*p* value
Number of patients (%)	34 (85%)	6 (15%)	
Gender			
Female n, (%)	21 (61.8%)	4 (66.7%)	0.6
Male *n*, (%)	13 (38.2%)	2 (33.3%)	
Mean age (SD)	34.7 (11)	39.5 (15)	0.36
Mean BMI at baseline (SD)	24.2 (12.9)	25.2 (12)	0.948
Mean fasting glucose (SD)	109 (30)	122 (40)	0.361
Mean Hba1C (SD)	5.9 (0.8)	7.5 (1.6)	0.518
Mean IGF-I at acromegaly diagnosis (SD)	3.3 (1.2)	4.8 (2.4)	0.01
Mean GH at acromegaly diagnosis (SD)	17 (8)	20 (10)	0.6
Invasion			
*Not-invasive*	11 (32.4%)	0	0.111
*Cavernous sinus*	20 (58.8%)	2 (33.3%)	
Cavernous sinus plus other structures*	3 (8.6%)	4 (66.7%)	
GHR isoform			
*flfl-carriers*	16 (64%)	3 (60%)	0.619
*d3-carries*	9 (36%)	2 (40%)	
SSTR2°			
*Score 0–1*	8 (44.4%)	3 (75%)	0.293
*Score 2–3*	10 (55.6%)	1 (25%)	
SSTR5°			
*Score 0–1*	2 (12.5%)	0	0.632
*Score 2–3*	14 (87.5%)	4 (100%)	
Mean Ki-67 (SD)	2.5 (1.5)	3.5 (2)	0.203
Resistance to first gen-SRLs			
*Partial n, (%)*	11 (32.5%)	0	0.179
*Complete n, (%)*	23 (67.5%)	6 (100%)	
Mean IGF-I at baseline (SD)	3.1 (1.9)	3.3 (1.7)	0.993
Mean GH at baseline (SD)	4 (3)	5 (2)	0.752

Univariate analysis

At the end of the study, 27 patients (67.5%) reached biochemical control of acromegaly. Glucose metabolism improved in 3 (7.5%), worsened in 26 (65%) and remained unchanged in 11 patients (27.5%). In particular, 6 patients were considered euglycemic (15%); 20 patients (50%) had IGT/IFG and 14 patients (35%) DM. Eleven patients (27.5%) were on treatment with low calorie low glycemic index diet, 14 patients (35%) with oral hypoglycemic drugs and 9 patients (22.5%) with insulin. The remaining 6 patients did not follow specific therapeutic indications (15%). The clinical features of the two treatment groups are summarized in Table 1.

### Glucose outcome and treatments

#### Pasireotide LAR

At baseline, glucose metabolism was considered normal in 17 patients (50%), 12 (35.3%) had IGT/IFG and 5 patients (14.7%) had DM. Nine patients (26.5%) were diet-controlled, 2 patients (5.9%) treated with oral hypoglycemic drugs and 4 patients (11.8%) with insulin. The remaining 19 patients did not require treatments for glucose abnormalities (55.9%) (Table 1). At the end of the study, glucose metabolism was unchanged in 9 (26.5%) and worsened in 25 patients (73.5%), at the end of the study.

**Fig. 1:**
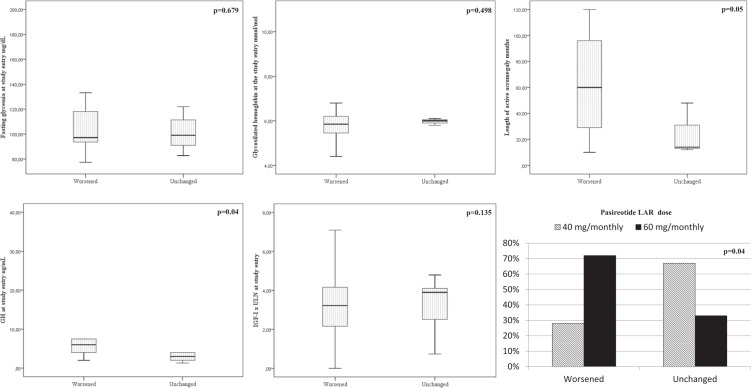
Factors associated with glucose metabolism outcome in patients on treatment with Pasireotide LAR. Box plots. Univariate analysis

In the PAS-LAR group, GH value at baseline was significatly higher (*p* = 0.04) in patients with worsening of glucose metabolism at the end of the study compared to patients with stable glucose status during the study period. No improvement of glucose metabolism was observed during PAS- LAR treatment (Fig. 1). Glucose metabolism outcome was similar among females and males (*p* = 0.6). Additionaly, we found that 12 of the 25 patients who experienced worsening of glucose metabolism had IGT/IFG or DM at baseline and the remaing 13 patients had normal glucose metabolism at baseline. Among these 13 patients, 5 patients were treated with oral hypoglicemic drugs and 2 patients with insulin (Table 2).

**Table 2:** Study population features stratified for acromegaly treatments

	Pasireotide LAR	Pasireotide LAR plus Peg-V	*p*-value
Glycemic assessment at baseline			
Normal glucose status *n*, (%)	17 (50%)	1 (16.7%)	0.019
IGT/IFG *n*, (%)	12 (35.3%)	1 (16.7%)	
Diabetes Mellitus *n*, (%)	5 (14.7%)	4 (66.4%)	
Treatment for glycemic assessment at baseline			
None *n*, (%)	19 (55.9%)	1 (16.7%)	0.258
Hypocaloric hypoglycemic diet *n*, (%)	9 (26.5%)	2 (18.2%)	
Oral hypoglycemic drugs *n*, (%)	2 (5.9%)	1 (16.7%)	
Insulin *n*, (%)	4 (11.8%)	2 (18.2%)	
Glycemic assessment at the end of study			
Normal glucose status *n*, (%)	5 (14.7%)	1 (16.7%)	0.651
IGT/IFG *n*, (%)	18 (52.9%)	2 (33.3%)	
Diabetes Mellitus *n*, (%)	11 (32.4%)	3 (50%)	
Treatment for glycemin control			
None *n*, (%)	5 (14.7%)	1 (16.7%)	0.766
Hypocaloric hypoglycemic diet *n*, (%)	9 (26.5%)	2 (18.2%)	
Oral hypoglycemic drugs *n*, (%)	13 (38.2%)	1 (16.7%)	
Insulin *n*, (%)	7 (20.6%)	2 (18.2%)	
Acromegaly at the end of study			
Controlled *n*, (%)	22 (64.7%)	5 (83.3%)	0.351
Active *n*, (%)	12 (35.3%)	1 (16.7%)	

Univariate analysis

HbA1c was higher in the group of biochemically active patients on treatment with higher dose of PAS- LAR (60 mg monthly) then in those on treatment with 40 mg/monthly, whereas the worsening in HbA1c was significantly greater with the higher dose in both patients with biochemically controlled and active acromegaly (Table 3).

**Table 3:** HbA1c and Δ HbA1c at the end of the study according to Pasireotide LAR dose

	Acromegaly disease status, number of patients (%)	Pasireotide Lar monthly dose	Pegvisomant daily dose	HbA1c	*p* value	Δ HbA1c	*p* value
Pasireotide Lar alone (34 pts)	Controlled, 22 pts (64.7%)	40 mg, 9 pts (40.9%)	Na	6.4 (0.4)	0.08	0.4 (0.2)	0.003
		60 mg, 13 pts (59.1%)		7.1 (1.3)		1.2 (1.4)	
	Active, 12 pts (35.3%)	40 mg, 4 pts (33.3%)		5.8 (0.9)	0.04	0.2 (0.2)	0.03
		60 mg, 8 pts (66.7%)		7.4 (2.8)		0.9 (2)	
Pasireotide Lar plus Peg-V (6 pts)	Controlled, 5 pts (83.3%)	60 mg, 5 pts (83.3%)	20 mg (SD: 5)	7.6 (1.5)	Na	−0.3 (1)	Na
	Active, 1 pt (16.7%)	60 mg, 1 pt (16.7%)	30 mg	5.8		0.4	

Δ HbA1c was calculated as the difference of HbA1c collected at the end of the study and that collected at baseline

#### Pasireotide LAR plus Pegvisomant

**Fig. 2:**
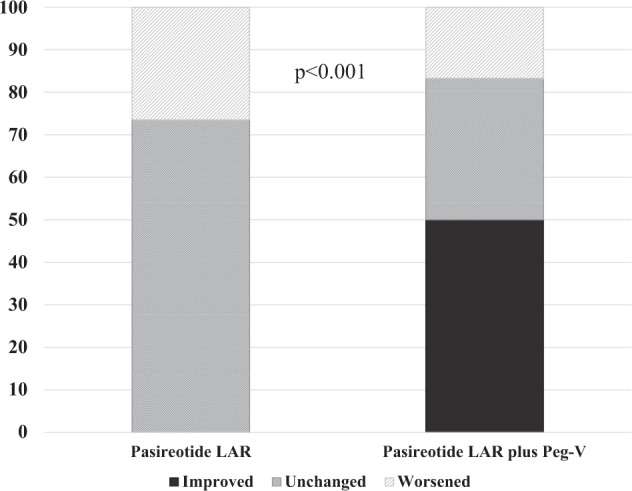
Glucose outcome according to acromegaly treatments. Histogram. Univariate analysis

**Fig. 3:**
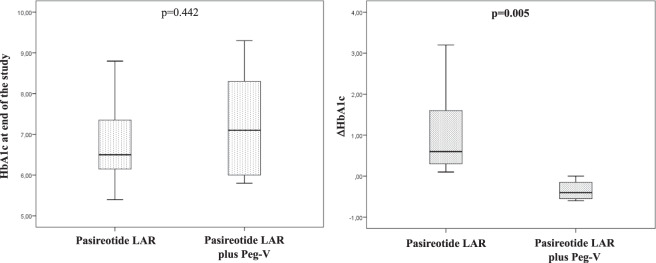
Glicometabolic control at the end of the study according to treatments. Box plot. Univariate analysis

Six patients were on treatment with combination therapy with Peg-V and PAS- LAR. Before starting this combination therapy, a single patient was affected by IGT/IFG (16.7%) and 4 patients by DM (66.4%). At the end of the study, glucose metabolism was unchanged in 2 (33.3%), worsened in a single patient (16.7%) and improved in 3 cases (50%). Therefore, combination treatment with PAS- LAR plus Peg-V was associated with a lower frequency of glucose metabolism worsening and with a higher frequency of glucose metabolism improving as compared to PAS- LAR alone (*p* < 0.001) (Fig. 2) and with a significantly reduction of HbA1c during the treatment, (*p* = 0.005) (Fig. 3).

## Discussion

Glucose homeostasis represents a relevant clinical problem in acromegaly, to which both GH and IGF-I hypersecrection and medical therapies can contribute, although their reciprocal role is not yet completely cLARified [2, 11–15, 21, 22]. Concerning the latter aspect, an increased awareness was raised by the PAS- LAR use, which was associated with higher diabetes risk [13–15].

In this study, we investigated the alterations of glucose metabolism in acromegaly patients before and during long-term treatment PAS- LAR alone and in combination with Peg-V.

Before starting treatment with PAS- LAR, 32.5% of patients had IGT/IFG and 22.5% had DM. These data are in line with previous epidemiological data and with the notion that conventional SRLs have only a marginal role in glucose homeostasis [23], as all the patients of this cohort were treated with conventional SRLs, before starting PAS- LAR. Instead, at the end of the study, the prevalence of IGT/IFG and DM increased, involving respectively the 50% and the 35% of cases. We found that the glucose status worsened in 25/34 on PAS-LAR (73.5%) and in a single patient treated with Pasi- LAR plus Peg-V. Our data are in line with previous authors that reported hyperglycemia and new onset DM in around 57% and 20% of patients on treatment with PAS- LAR [13–15]. Interestingly, more than half of patients with worsened glusose metabolism had normal glycometabolic status before treatment although it was previously suggested a predictive role of pre-PAS LAR treament glucose parameters [24]. Conversely, we found that the higher PAS-LAR dose (60 mg/monthly) correlated significantly with glucose control worsening, in particular with the increase in HbA1c, both in active and controlled acromegaly, at the end of the study. Another original finding of our study is that the higher GH levels may predict the worsening of glucose metabolism during Pasi-LAR treatment. This finding was identified also in other co-morbidities of acromegaly as bone fragility [25–28]. GH and IGF-I induce several metabolic changes that are actually not completely clarified [29, 30]. GH acts with a bimodal effect on glucose homeostasis, stimulating beta-cell proliferation, insulin synthesis and secretion, but also increasing lipolysis, gluconeogenesis [31, 32] and inducing systemic insulin resistance in active acromegaly [33, 34]. As a consequence, GH excess per se may induce IFG, IGT, and DMII [35, 36]. However, GH action is also mediated by IGF-I that is mostly synthesized in hepatocytes upon GH receptor binding [37]. Conversely, IGF-I has been shown to increase insulin sensitivity [29]. Deterioration of glucose metabolism in patients with higher GH and IG-I levels has also been observed in patients partially resistant to SRL treated with high octreotide dose [38].

Taken together, these findings suggest that the higher dose of PAS-LAR needed to control a more biochemically active disease may have contributed to a greater glucose metabolism alteration. In fact, it is already known that in the presence of a more active disease at baseline, it is more difficult to obtain biochemical control of acromegaly [39]. Moreover, PAS-LAR has been shown to decrease insulin secretion acting at pancreatic beta cell level and to also decrease GlP and GLP-1 secretion [24]. Therefore, based on our data it can be hypothesized that in our population a combined mechanism represented by negative direct pancreatic effect of PAS-LAR combined with detrimental effects of high GH on insulin resistance may occur [40] although our data did not allow to clarify which of the two mechanisms could be cliinically prevalent in determining the observed metaboslic worsening.

**Fig. 4:**
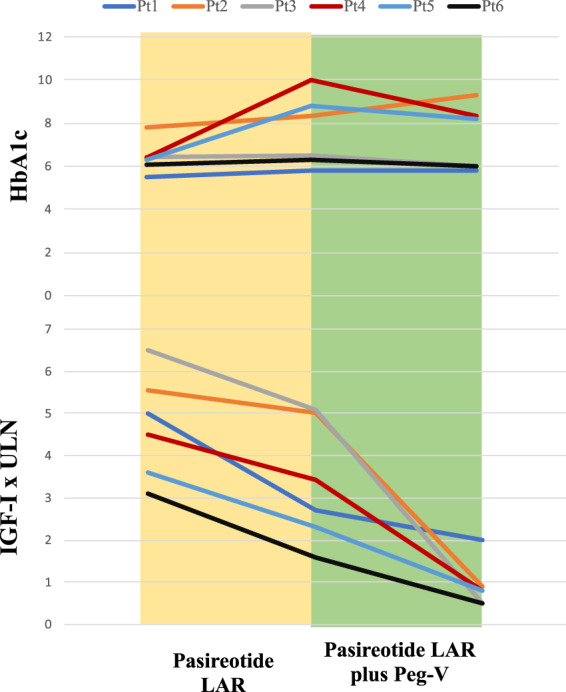
Trend of HbA1c and IGF-I during treatment with Pasireotide LAR as monotherapy and in combination with Peg-V

Finally, we found in the group of patients under Peg-V plus PAS- LAR the combination treatment may not only improve biochemical control but also mitigate the worsening of glucose metabolism caused by PAS- LAR alone. In fact, as shown in Fig. 4, all but one patients experienced a reduction of the HbA1c. Interestingly, the reduction of pre-treatment HbA1c was significantlly greater in patients treated with PAS-LAR in combination with Peg-V as compared to those of patients treated with PAS-LAR as monotherapy (Fig. 3). These results are different with those of Muhammad et al and could be explained by uptitrating doses of Peg-V in a clinical setting like ours vs a prospective design with fixed doses which aimed to spare Peg-V dose. Furthermore, PAS- LAR dose in that study was 60 mg monthly which we showed to have a worsening effect on glucose in our study.

This improvement of glucose metabolism can also be due to the biochemical efficacy of this combination therapy, that we recently described to be effective in a group of acromegaly patients affected by a disease resistant to multimodal treatments [41]. In fact, patients who required combination treatment PAS- LAR plus Peg-V had higher values of IGF-I at acromegaly diagnosis, if compared with cases treated with PAS- LAR alone. However, these IGF-I levels did not correlate with the glucose outcome (*p* = 0.943). Alternatively, though not confirmed in Muhammad et al. [42], it may be suggested that Peg-V per se may have a protective effect on the hyperglycemic effect of PAS- LAR possibly reducing insulin resistance in some patients [43].

The significant limitations of this study are its retrospective design and the absence of randomization for the acromegaly treatment. Likewise, the small size of the group of patients treated with PAS- LAR plus Peg-V reduced the power of comparative analysis. However, although the number of patients by us reported on treatment with PAS-LAR in combination with Peg-V is limited, experiences on glucose outcomes with this new form of therapy are scant and controversial [21, 38, 42, 44]. This combination is also currently included in consensus guidelines [21] for particularly resistant acromegaly, thus we think that this multicentric experience can be a useful addition to the available clinical evidence. In fact, based on our data it can be hypothesized that in patients in whom residual tumor and diabetes are both clinical concerns [45] and are not sufficiently controlled by first generation SRLs, a combination of PAS-LAR and Peg-V may be a viable option. In addition, the design of this study did not allow us to compare head to head Peg-V vs PAS-LAR in their effects on glucose metabolsim and did not provide any direct insights on the mechanisms underlying their effects.

In conclusion, we confirm previous data that glucose metabolism is often worsened during second line medical treatment with PAS-LAR in acromegaly. Our results suggest that this glucose worsening may be due to the use of PAS-LAR according to guidelines (45) in acromegaly patients with severe disease which in turn require high doses of the drug. In fact, both high GH levels per se as well as PAS-LAR may directly and potentially synergistically be detrimental for glucose metabolism. In this light, our data, although limited by small numbers, are among the first to suggest that the combination treatment PAS-LAR plus Peg-V can not only improve biochemical control but also glucose homeostasis in selected patients. If confirmed in larger studies, this combination could be suggested earlier in the treatment of patients with resistant acromegaly and concomitant abnormalities in glucose metabolism.
